# Dhcr7 Regulates Palatal Shelf Fusion through Regulation of Shh and Bmp2 Expression

**DOI:** 10.1155/2016/7532714

**Published:** 2016-03-15

**Authors:** Wen-lin Xiao, Dai-zun Zhang, Hong Xu, Cui-zhu Zhuang

**Affiliations:** ^1^Department of Stomatology, The Affiliated Hospital of Qingdao University, Qingdao 266003, China; ^2^The Key Laboratory of Oral Clinical Medicine of Shandong Province, The Affiliated Hospital of Qingdao University, Qingdao 266003, China; ^3^Department of Oral and Maxillofacial Surgery, The Affiliated Hospital of Qingdao University, Qingdao 266003, China

## Abstract

The aim of this study was to investigate the effect of the 7-dehydrocholesterol reductase (*Dhcr7*) gene and identify signaling pathways involved in regulation of embryonic palatogenesis. The expression of* Dhcr7* and its protein product were examined during murine normal embryonic palatogenesis via a reverse transcription polymerase chain reaction (RT-PCR) and Western blot (WB). RNA interference (RNAi) technology was used to inhibit* Dhcr7* expression in a palatal shelf culture* in vitro*. The effects of Dhcr7 on palatogenesis and palatal fusion were examined by scanning electron microscopy (SEM). The expression changes of Dhcr7, Sonic Hedgehog (Shh), and bone morphogenetic protein-2 (Bmp2) were measured by RT-PCR and WB after* Dhcr7* gene silencing and the addition of exogenous cholesterol. The results showed that the palatal shelf failed to complete normal development and fusion when* Dhcr7* expression was inhibited. The inhibitory effect study of RNAi on the development of the palatal shelf supported that cholesterol supplementation did not alter the silencing of Dhcr7. Shh and Bmp2 expressions were reduced after* Dhcr7* gene silencing, and administration of exogenous cholesterol did not affect Dhcr7 expression; however Shh and Bmp2 expressions increased. We conclude that* Dhcr7* plays a role in growth of the palatal shelf and can regulate palatogenesis through alterations in the levels of Shh and Bmp2.

## 1. Introduction

Secondary palate development in the mouse embryo begins at gestation day 11.5 (GD11.5), with a tissue fold at the palatal location in the oral region, and mesenchymal cell proliferation in the maxillary processes that form the palatal shelf, primordium. The palatal shelf grows vertically along both sides of the tongue between GD12.5 and GD13.5. Both sides of the palatal shelf elevate above the horizontal position of the tongue from GD13.5 to GD14.5 and then fuse to form a continuous palate on GD15.5 [1]. The growth and development of a normal mammalian secondary palate requires fine collaboration between morphogenetic factors and mesenchymal cells. Genetic and environmental factors can lead to the occurrence of cleft palate at every stage of palatal development. Cleft lip and palate is one of the most common congenital malformations in the oral and maxillofacial region, and it can adversely impact all components of maxillofacial function, except vision [2].

The human* Dhcr7* gene is mapped to 11q12-13. A mutation in* Dhcr7* leads to a decline in 7-dehydrocholesterol reductase activity, lowering cholesterol levels, while its precursor 7-dehydrocholesterol (DHC) accumulates. This leads to the possibility of multiple growth and development deformities, including cleft palate deformities, known as Smith-Lemli-Opitz syndrome (SLOS) [3]. Cholesterol metabolism disorders affect the Sonic Hedgehog (Shh) pathways [4]. The* Shh* gene plays an important role in embryonic development, including palatal shelf development.

The specific physiological and pathological processes of* Dhcr7* are not fully understood. In C57BL/6J mice, we studied the role of* Dhcr7* in palatal shelf development and sought to find signaling pathways involved in regulating it.

## 2. Materials and Methods

### 2.1. Palatal Shelf Organ Culture

C57BL/6J inbred strains of mice (Beijing, HuaFuKang Experimental Animal Center, China) were maintained at a temperature of 22°C with an alternating light/dark cycle and were provided access to food and filtered water. Mature (8-week-old) mice were mated overnight and the presence of a vaginal plug was taken the following morning as evidence of mating (gestation day 0.5, GD 0.5). The GD13.5 mice were sacrificed and placed in 75% ethanol for 1 min. The embryos were removed under aseptic conditions and the head of the mouse embryo was sheared by eye scissors and placed in sterile solution in Ca^++^/Mg^++^-free phosphate buffered saline (CMF-PBS) and washed three times. The suspension culture method of Shiota [5] was used. The fetal head was placed in an inverted position in a stereo microscope and the jaw and tongue body were resected from the horizontal left and right mouth corners, with the bilateral palatal shelf exposed. The head above the eyes was removed by horizontal incision. The bilateral palatal shelf and part of the upper jaw were placed in 35 mm petri dishes and cultured in 0.5 mL serum-free DMEM (Gibco) supplemented with 2 mM L-glutamine (Gibco), and 0.1 mM nonessential amino acids (Sigma) in an incubator at 37°C on a roller device at 25 rpm for up to 48 hours with a mixture of 95% air/5% CO_2_.

### 2.2. Adenovirus Production

Adenovirus was produced following the AdEasy protocol. Briefly, four siRNA sequences targeting the* Dhcr7* gene in mice were designed according to the method of Incardona and Roelink [6]. Embryonic palatal mesenchymal (EPM) cells of C57BL/6J were used to select the most effective siRNA sequence. And then, the most effective shRNA sequence (sense strand: 5′-TCGA CCA ACT ACG TGT TAG ACT T GAGTACTG AAG TGT AAC ACG TAGM ATG G TTTTT-3′; antisense strand: 5′-CTAG AAAAA CCA ACT ACG TGT TAG ACT T CAGTACTC AAG TGT AAC ACG TAGM ATG G-3′) and scrambled shRNA sequence (sense strand: 5′-TCGA TTC TCC GAA CGT GTC ACG T GAGTACTG ACG TGA CAC GTT CGG AGA A TTTTT-3′; antisense strand: 5′-CTAG AAAAA TTC TCC GAA CGT GTC ACG T CAGTACTC ACG TGA CAC GTT CGG AGA A-3′) were synthesized (Shanghai Gemma Pharmaceutical Technology Company) and cloned into a pAdTrack-CMV vector (Agilent Technologies) at Xho I and Xba I restriction enzyme sites. The* AdsiDhcr7* adenovirus expression plasmid was generated from recombination between the pAdEasy-1 vector and pAdTrack-CMV-*siDhcr7* in BJ5183 competent cells (Agilent Technologies). The Ad*siDhcr7* plasmid was linearized with Pac I restriction enzyme and subsequently transfected into AD293 cells using Lipofectamine 2000 reagent (Invitrogen) according to the manufacturer's protocol for adenovirus packaging. Adenoviruses were harvested at 14 to 20 days after transfection.

### 2.3. *Dhcr7* siRNA Adenovirus Virus Infection

Briefly, palatal shelves were dissected from GD13.5 C57BL/6 mouse embryos using microscissors, placed on a 0.8 *μ*m pore size filter (Merck Millipore) in 35 mm culture dishes in a 37°C incubator with 5% CO_2_. The GD13.5 palatal shelves in organ culture were grouped into (A) normal control group: cholesterol-free DMEM/F12 medium (Hyclone) + cultured palatal shelves; (B) control empty adenovirus group: cholesterol-free MEM/F12 medium + cultured palatal shelves + control empty adenovirus; and (C) experimental group: cholesterol-free DMEM/F12 medium + cultured palatal shelves +* Dhcr7* siRNA adenovirus. Every group included 10 individual mice. 5.1 × 10^7^ Relative Infection Units/mL (RIU/mL) of adenovirus were added to the 35 mm culture dish according to the group and incubated for another 24 hours. Subsequently, the palatal shelves were harvested and* Dhcr7* and its protein in all the palatal shelves were analyzed by RT-PCR and Western blot. Scanning electron microscopy was also performed.

### 2.4. Exogenous Cholesterol Supplementation

In the preliminary experiments, the medium including* Dhcr7* siRNA was changed to cholesterol-free DMEM/F12 medium after 12 h. Cholesterol (Sigma) was subsequently added at 0 ng/mL, 200 ng/mL, 400 ng/mL, and 600 ng/mL. The concentrations of cholesterol chosen were derived from the work of Hayavi and Halbert [7] and Wassif et al. [8]. These preliminary results revealed that* Dhcr7* silencing caused a failure of fusion of the cultured palatal shelves that was successfully reversed by exogenous cholesterol. The chance of fusion in cultivated palatal shelves increased directly with exogenous cholesterol concentration, where cultivated palatal shelves were completely fused with 600 ng/mL of exogenous cholesterol. We subsequently designed another experiment in which GD13.5 palatal shelves were placed in organ culture and grouped into three groups of 10 murine specimens each: (A) normal control group: cholesterol-free DMEM/F12 medium + cultured palatal shelves, changing medium every 24 hours, cultivated for a total of 48 hours; (B) gene silencing group: cholesterol-free DMEM/F12 medium + cultured palatal shelves +* Dhcr7* siRNA adenovirus, the media being replaced with cholesterol-free DMEM/F12 medium 48 hours after transfection, cultivated for a total of 48 hours; and (C) cholesterol supplementation group: cholesterol-free DMEM/F12 medium + cultured palatal shelves +* Dhcr7* siRNA adenovirus, the media being replaced with DMEM/F12 medium with 600 ng/mL cholesterol 24 hours after transfection, cultivated for a total of 48 hours. The* Dhcr7*,* Shh*, and* Bmp2* in all palatal shelves were analyzed by RT-PCR and their proteins verified by Western blot.

### 2.5. RT-PCR

A pair of palatal shelves were dissected using a stereo microscope from cultured palatal organ tissue, and the total RNA of the cells of palatal shelves was isolated using Trizol (Takara), and a PrimeScript RT-PCR Kit (Takara) was subsequently used to performed the PCR: 2.5 mL cDNA, 1x PCR buffer (AMS), 200 mM dNTPs, 0.2 mM of each primer pair, and 1 unit/25 mL reaction Taq DNA polymerase (Takara). Gene sequences of the primers are shown in Table 1.

### 2.6. Western Blot Analysis

Western blot analysis was performed to assess the protein expression of Dhcr7, Shh, and Bmp2. The palatal shelf cells were harvested at different time points for protein extraction with the M-PER mammalian protein extraction reagent (Pierce). Equal amounts of total protein were loaded onto a 10% SDS-PAGE gel and transferred onto a PVDF membrane (Millipore) in a Trans-Blot SD Semi-Dry Electrophoretic Transfer Cell (Bio-Rad) at 15 V for 30 min. The membrane was blocked for 2 h at room temperature with 5% skim milk in Tris-buffered saline containing 0.05% Tween-20 (TTBS) and incubated overnight with antibodies (see below). Proteins were then incubated with a peroxidase-conjugated secondary antibody for 1 h and developed with an ECL1 Detection kit (Amersham), according to the manufacturer's instructions. The protein was detected with an ECL system (Amersham) by chemoluminescence and visualized on radiographic film. Protein expression was quantified with Gel-Pro Analyzer 3.1 software (Media Cybernetics). The antibodies used in the present study were Dhcr7 antibody (1 : 2,000, Abcam), Shh antibody (1 : 4,000, Abcam), and Bmp2 antibody (1 : 5,000, Abcam). Monoclonal anti-*β*-actin antibody (Sigma), diluted 1 : 5000, was used as a loading control.

### 2.7. Scanning Electron Microscopy (SEM)

For visualization of the fine surface structure of palatal shelves, we carried out SEM as described by Abbott et al. [9]. Samples were fixed for 2 h in 0.025 g/mL glutaraldehyde solution at room temperature and were subsequently dehydrated through a graded series of ethanol, isoamyl acetate replacement, critical point drying, and gold plated in a coater (JFC-1600, JEOL). The images were viewed on a Zeiss DSM 950 SEM.

### 2.8. Statistical Analysis

At least three assays, each in triplicate, were performed. Grey values of RT-PCR and WB target bands were analyzed by Quantity One software (Bio-Rad); SPSS18.0 statistical software was used for analysis. Data were analyzed by normality and homogeneity of variance tests. A single factor ANOVA was used in the overall tests and *t*-test was used in the two group tests.

## 3. Results

### 3.1. *Dhcr7 *siRNA Adenovirus Inhibited* Dhcr7* Expression in Palatal Culture

**Figure 1:**
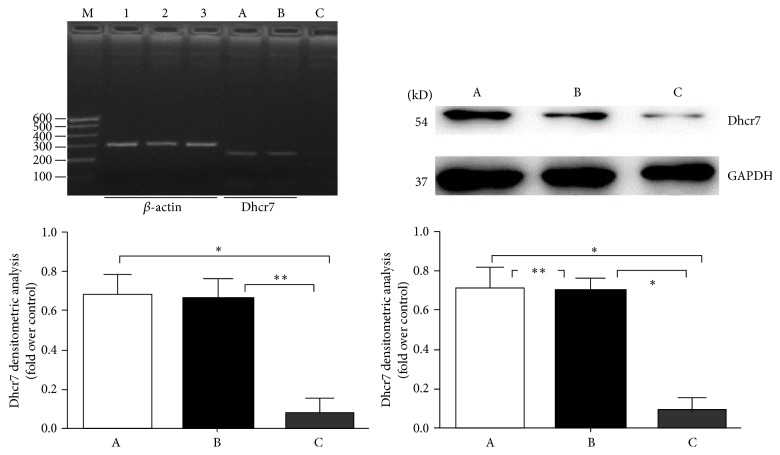
The Dhcr7 mRNA and protein expression levels of the different treatment groups. In the left image and graph, RT-PCR results revealed significant differences when group A or B was compared with group C (^*∗*^ *P* < 0.05, ^*∗∗*^ *P* < 0.05); the difference between groups A and B was not statistically significant (*P* > 0.05). In the right image and graph, WB analysis showed that the ratio of Dhcr7/GAPDH difference was statistically significant when group C was compared with group A or B (^*∗*^ *P* < 0.05); the difference was not statistically significant when group A was compared with group B (^*∗∗*^ *P* > 0.05). In the graphs, 1, 2, and 3 show the expression of *β*-actin; ABC show the normal control group, control empty adenovirus group, and the experimental group, respectively, where A is normal control group, B is control empty adenovirus group, and C is experimental group.

RT-PCR for the Dhcr7 mRNA expression of the normal control group, control empty adenovirus, and experimental groups revealed that the experimental group* Dhcr7* mRNA expression (0.090 ± 0.057) was significantly less than the normal control group (0.692 ± 0.051) and control empty adenovirus group (0.683 ± 0.027). The difference was statistically significant (^*∗*^ *P* < 0.05; ^*∗∗*^ *P* < 0.05) (Figure 1).

WB for Dhcr7 revealed similar protein expression in the normal control group (0.712 ± 0.097) and control empty adenovirus group (0.698 ± 0.065) (^*∗∗*^ *P* > 0.05). However, expression in the experimental group (0.087 ± 0.065) decreased dramatically (^*∗*^ *P* < 0.05) (Figure 1).

### 3.2. Inhibition of* Dhcr7* Expression by siRNA Adenovirus Blocked Palatal Fusion

**Figure 2:**
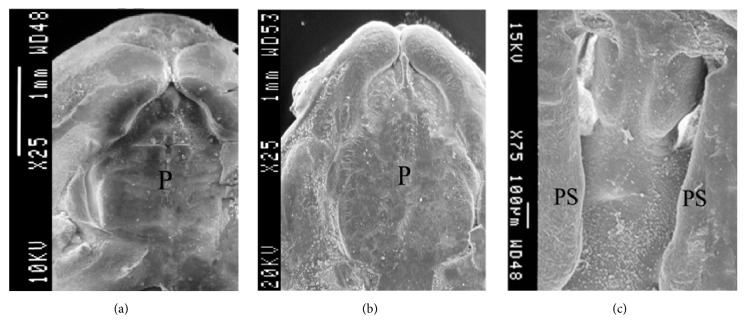
SEM of palatal fusion cases. Normal control (a), control empty adenovirus (b), and an experimental specimen (c). P: palate; NC: nasal cavity.

Palatal fusions were evaluated by SEM. When the normal control group and control empty adenovirus group palates were cultured for 48 h, both sides of the palate contacted and fused (Figures 2(a) and 2(b)). In the experimental group, a substantial defect remained between the two sides of palate, with the lower nasal cavity structures visible (Figure 2(c)).

### 3.3. Addition of Exogenous Cholesterol Does Not Affect* Dhcr7* Silencing

**Figure 3:**
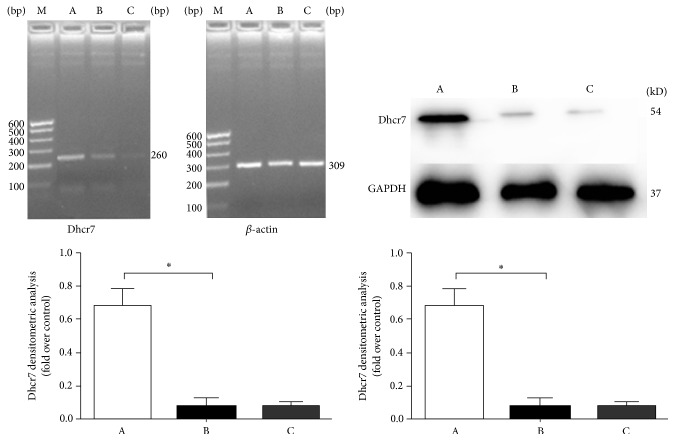
Dhcr7 gene mRNA (left image and graph) and protein (right image and graph) expression analysis. A: control group; B: Dhcr7-siRNA inhibited group; C: supplemental cholesterol group. RT-PCR detection indicates that the difference is statistically significant when group B was compared with group A (^*∗*^ *P* < 0.05); the difference was not statistically significant when group C was compared with group B (*P* > 0.05). WB analysis showed that the ratio of Dhcr7/GAPDH difference was statistically significant when group B is compared with group A (^*∗*^ *P* < 0.05); the difference is not statistically significant when group C is compared with group B (*P* > 0.05).

Experiments to determine the effect of cholesterol supplementation on Dhcr7 silencing were performed. The mRNA and protein levels from the control group (group A),* Dhcr7*-siRNA experimental group (group B), and the cholesterol treated group (group C) were analyzed. In group A and group B, the Dhcr7 mRNA expression was 0.691 ± 0.101 and 0.083 ± 0.045, respectively, and Dhcr7 protein expression of the two groups was 0.673 ± 0.081 and 0.102 ± 0.064, respectively.* Dhcr7* mRNA and protein expression decreased after* Dhcr7* silencing in group B by 87.8% and 84.6%, respectively, compared to group A (^*∗*^ *P* < 0.05 for both);* Dhcr7* mRNA (0.074 ± 0.034) and protein (0.137 ± 0.045) expression in group C were almost unchanged compared with group B (*P* > 0.05) (Figure 3), supporting that cholesterol supplementation did not alter the silencing of* Dhcr7*.

### 3.4. Effects of Adding Exogenous Cholesterol on Shh Expression after* Dhcr7* Silencing

**Figure 4:**
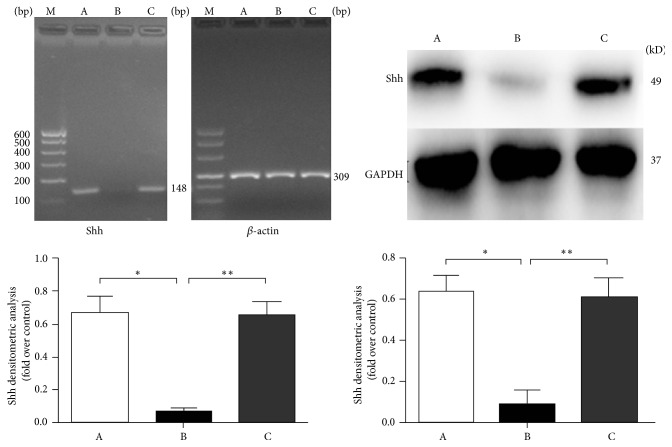
Shh gene mRNA (left image and graph) and protein (right image and graph) expression analysis. A: control group; B: Dhcr7-siRNA inhibited group; C: supplemental cholesterol group. RT-PCR detection indicates that the difference was statistically significant when group B was compared with group A (^*∗*^ *P* < 0.05); the difference was also statistically significant when group C was compared with group B (^*∗∗*^ *P* < 0.05). WB analysis showed that the ratio of Shh/GAPDH difference was statistically significant when group B was compared with group A (^*∗*^ *P* < 0.05); the difference was statistically significant when group C was compared with group B (^*∗∗*^ *P* < 0.05).

mRNA and protein levels from the blank control group (A),* Dhcr7*-siRNA experimental group (B), and cholesterol supplementation group (C) were extracted and analyzed for Shh. In group A and group B, the* Shh* mRNA expression was 0.667 ± 0.093 and 0.063 ± 0.018, respectively, and* Shh* protein expression of the two groups was 0.642 ± 0.050 and 0.113 ± 0.029, respectively. mRNA and protein expression levels in group B were reduced by 89.3% and 79.5%, compared with group A (^*∗*^ *P* < 0.05 for both comparisons). In group C,* Shh* mRNA (0.649 ± 0.085) and protein (0.628 ± 0.033) expression levels were upregulated by 90.3% and 82.0%, compared with group B (^*∗∗*^ *P* < 0.05), supporting that cholesterol supplementation effectively reversed the effect of* Dhcr7* silencing on Shh expression (Figure 4).

### 3.5. Effects of Adding Exogenous Cholesterol on Bmp2 Expression after* Dhcr7* Silencing

**Figure 5:**
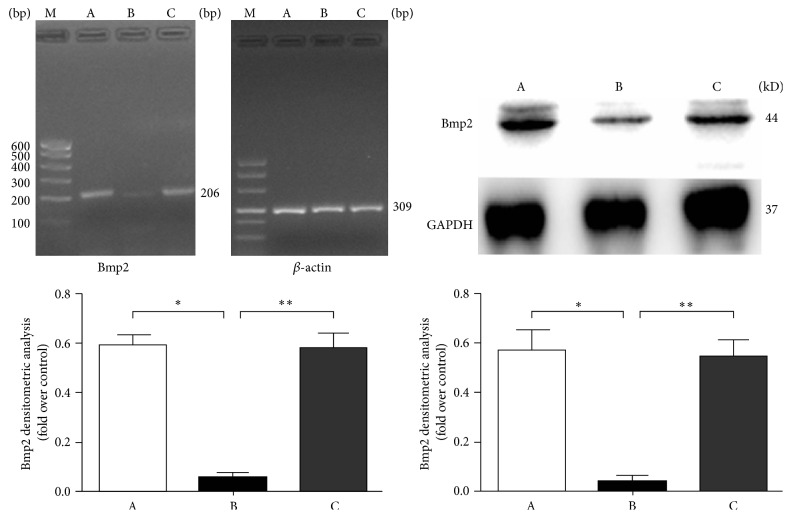
Bmp2 gene mRNA (left) and protein (right) expression analysis. A: control group; B: Dhcr7-siRNA inhibited group; C: supplemental cholesterol group. RT-PCR detection indicated that the difference was statistically significant when group B was compared with group A (^*∗*^ *P* < 0.05); The difference was also statistically significant when group C was compared with group B (^*∗∗*^ *P* < 0.05). WB analysis showed that the ratio of Bmp2/GAPDH difference is statistically significant when group B is compared with group A (^*∗*^ *P* < 0.05); the difference is statistically significant when group C compared with group B (^*∗∗*^ *P* < 0.05).

The mRNA and protein levels from the blank control group (A),* Dhcr7*-siRNA experimental group (B), and cholesterol supplementation group (C) were extracted and analyzed for Bmp2 expression. In group A and group B, the* Bmp2* mRNA expression was 0.591 ± 0.043 and 0.054 ± 0.018, respectively, and* Bmp2* protein expression levels in the two groups were separately 0.582 ± 0.037 and 0.046 ± 0.029, respectively.* Bmp2* mRNA and protein expression levels in group B were reduced by 88.6% and 87.8%, compared with group A (^*∗*^ *P* < 0.05). In group C,* Bmp2* mRNA (0.578 ± 0.032) and protein (0.577 ± 0.025) expression levels were upregulated by 88.2% and 87.5% compared with group B (^*∗∗*^ *P* < 0.05). These data support that cholesterol supplementation preserved Bmp2 expression that would otherwise be reduced by* Dhcr7* silencing (Figure 5).

## 4. Discussion

GD13.5–GD15.5 days are the critical period in development of the mouse embryonic palate. Disturbance of any of the developmental events can lead to the occurrence of cleft palate [10]. There is active research on how gene expression affects palate growth and palatal fusion at this stage. In a cultured embryonic mouse palate model, we altered* Dhcr7* gene expression from GD13.5 to GD15.5 to evaluate the role of the* Dhcr7* gene in the development of the palate. The* Dhcr7* gene is normally expressed in palatal mesenchymal cells. Dhcr7 is mainly expressed in the palatal position nearest to the maxillary protrusion growth and development centers. The embryonic palatal tissue located on both sides of the tongue undergoes vertical growth on GD13.5. Both sides of the palate elevate and fuse on GD14.5.* Dhcr7* is then found to be expressed throughout the palate shelf. On GD15.5, the palatal medial epithelial seam (MES) disappears, and palatal fusion is complete.* Dhcr7* mRNA and protein are expressed in the embryonic palate from GD13.5 to GD15.5, with the highest expression levels on GD13.5 and GD14.5. The expression is significantly reduced on GD15.5.

Cholesterol metabolism disorders are a cause of Smith-Lemli-Opitz syndrome (SLOS) [3]. SLOS is an autosomal recessive genetic malformation syndrome consisting of craniofacial limb defects, forebrain deformities, and other midline developmental malformations. About 50% of patients present with cleft palate [11, 12]. Molecular genetic studies of the* Dhcr7* gene indicate that the most common mutations include IVS8-1G→C, as well as W151X, T93M, R404C, and V326L [13–15]. The human* Dhcr7* gene is located on 11q12-13. Because* DHCR7* mutations will lead to 7-dehydrocholesterol reductase activity loss, cholesterol biosynthesis is inhibited, cholesterol levels decrease, and precursor 7DHC (7-dehydrocholesterol) and others accumulate* in vivo*, which are thought to contribute to cleft palate and the other developmental abnormalities [3]. In SLOS patients [16] and in* Dhcr7* knockout (*Dhcr7*−/−) mouse models [8], cholesterol has been found to be low and 7DHC elevated in plasma and tissues.

There are many methods to suppress the expression of endogenous Dhcr7, but siRNA allows for silencing of specific genes at a specific period or a point in time. siRNA has been used in cell culture [17, 18] and organ culture [19, 20]. In our study, we used siRNA-mediated RNAi, designed for mice. Dhcr7 specific siRNA sequences were transfected, and RT-PCR and WB were used to detect the transfection efficiency. The results revealed a significant decrease in the expression of* Dhcr7 *mRNA and protein.

The role of cholesterol in embryonic development requires additional investigation [21]. In this study, inhibition of the expression of endogenous Dhcr7 allowed us to observe its effect on the growth and integration of the palate and to study the effect of the addition of exogenous cholesterol after such suppression. Our results demonstrated that inhibition of Dhcr7 can lead to palatal development failure, where the palatal sides cannot successfully contact and fuse. SEM visualization showed that both sides of the palate contacted and fused in both the normal control group and the control empty adenovirus group after the palates were cultured for 48 h. In the experimental group, a substantial defect remained between the two sides of the palate, with the lower nasal cavity structures visible. However, addition of exogenous cholesterol (600 ng/mL) can reverse this palatal formation failure so as to promote the development of palatal contact and fusion. Dhcr7 appears to play a direct role in palatal fusion. In the setting of Dhcr7 dysfunction, the catalytic synthesis of endogenous cholesterol cannot meet the needs for normal palate fusion, leading to the occurrence of cleft palate. Alcohol intake may affect cholesterol metabolism and during pregnancy may cause fetal craniofacial abnormalities [22]. The* DHCR7* gene is involved in cholesterol metabolism and has a mutation rate of 3%-4% [23]. Our current study provides some theoretical guidance for predicting and preventing the occurrence of cleft palate.

Recent studies have found an increasing number of genes playing an important role in palate development. These genes include transcription factors and growth factors and their receptors. Mutations can lead to the occurrence of cleft palate [24, 25]. The palate originates from migratory neural crest cells and differentiates into the mouth, nose, and pharynx in the cranial ectodermal ridge epithelium. As in other vertebrates, the mouse secondary palate development also depends on the interaction of mesenchymal and epithelial cells [26]. Peptide growth factors serve as signaling molecules [27]. Many polypeptide growth factors are involved in the development of the vertebrate maxillofacial structures. These factors include Shh, bone morphogenetic proteins (BMPs), and the transforming growth factor *β* (TGF*β*) superfamily [28]. During the development of chicken maxillofacial primordia, Shh is an indispensable peptide growth factor. Blocking expression of the Shh signaling peptide resulted in maxillofacial primordia growth inhibition. Shh overexpression promoted the growth of maxillofacial primordia by promoting cell proliferation [29]. Augmented BMP2, BMP4, and BMP7 expression accelerates cell proliferation in the maxillary processes [30, 31].

Shh plays a key role in the development of the central nervous system and facial structure as well as limb formation. Altering the cholesterol balance changes lipid raft stability and protein composition and can interfere with Shh signaling pathways. This can cause a variety of deformities, including cleft palate [32, 33]. Studies have found that Shh is expressed at a critical stage in the development of the palate in mice and is predominantly expressed in the palatal epithelial ridge [34]. Shh knockout mice present defects of the forebrain, notochord and limbs, and severe cleft palate [35]. This phenotype is similar to Dhcr7−/− mice [3]. Bmp2 is also expressed at a critical stage in the development of palate in mice [36]. It has been shown that Shh can induce Bmp2 expression in the palatal mesenchyme. Shh and Bmp2 induce mesenchymal proliferation [37] and promote palatal development.

## 5. Conclusion

In conclusion, our study demonstrates that* Dhcr7* regulates palatal fusion through participation in the Shh and Bmp2 signaling pathways.

## Figures and Tables

**Table 1:** Sequences of primers.

Primer	Size (bp)	Sequence (5′-3′)
Dhcr7	260 bp	5′-TTT CCT GCT GCT CTT CGC TC-3′
		3′-CTT GGA CGC CTC CCA CAT AA-5′

Shh	148 bp	5′-ATT TTC CAA TGT AAT AGC CGT CTT C-3′
		3′-CTG TCT TAC CTT CTT GAG ACA ATA A-5′

Bmp2	206 bp	5′-GGA CGT AGC CTC CCC AGG-3′
		3′-GCC TCG TGT CAG AAT GGG AG-5′

*β*-actin	309 bp	5′-GAA CCC TAA GGC CAA CC-3′
		3′-TGT CAC GCA CGA TTT CC-5′

## References

[1] Burdett D. N., Waterfield J. D., Shah R. M. (1988). Vertical development of the secondary palate in hamster embryos following exposure to 6-mercaptopurine. *Teratology*.

[2] Mossey P. A., Little J., Munger R. G., Dixon M. J., Shaw W. C. (2009). Cleft lip and palate. *The Lancet*.

[3] Yu H., Patel S. B. (2005). Recent insights into the Smith-Lemli-Opitz syndrome. *Clinical Genetics*.

[4] Gruchy N., Bigot N., Jeanne Pasquier C. (2014). Involvement and alteration of the Sonic Hedgehog pathway is associated with decreased cholesterol level in trisomy 18 and SLO amniocytes. *Molecular Genetics and Metabolism*.

[5] Takahara S., Takigawa T., Shiota K. (2004). Programmed cell death is not a necessary prerequisite for fusion of the fetal mouse palate. *International Journal of Developmental Biology*.

[6] Incardona J. P., Roelink H. (2000). The role of cholesterol in Shh signaling and teratogen-induced holoprosencephaly. *Cellular and Molecular Life Sciences*.

[7] Hayavi S., Halbert G. W. (2005). Synthetic low-density lipoprotein, a novel biomimetic lipid supplement for serum-free tissue culture. *Biotechnology Progress*.

[8] Wassif C. A., Zhu P., Kratz L. (2001). Biochemical, phenotypic and neurophysiological characterization of a genetic mouse model of RSH/Smith-Lemli-Opitz syndrome. *Human Molecular Genetics*.

[9] Abbott B. D., Hill L. G., Birnbaum L. S. (1990). Processes involved in retinoic acid production of small embryonic palatal shelves and limb defects. *Teratology*.

[10] Dhulipala V. C., Welshons W. V., Reddy C. S. (2006). Cell cycle proteins in normal and chemically induced abnormal secondary palate development: a review. *Human and Experimental Toxicology*.

[11] Porter F. D. (2006). Cholesterol precursors and facial clefting. *The Journal of Clinical Investigation*.

[12] Jezela-Stanek A., Ciara E., Malunowicz E. M. (2008). Mild Smith-Lemli-Opitz syndrome: further delineation of 5 Polish cases and review of the literature. *European Journal of Medical Genetics*.

[13] Ko J. S., Choi B. S., Seo J. K. (2010). A novel DHCR7 mutation in a Smith-Lemli-Opitz syndrome infant presenting with neonatal cholestasis. *Journal of Korean Medical Science*.

[14] Porter F. D. (2008). Smith-Lemli-Opitz syndrome: pathogenesis, diagnosis and management. *European Journal of Human Genetics*.

[15] Correa-Cerro L. S., Wassif C. A., Waye J. S. (2005). DHCR7 nonsense mutations and characterisation of mRNA nonsense mediated decay in Smith-Lemli-Opitz syndrome. *Journal of Medical Genetics*.

[16] Witsch-Baumgartner M., Schwentner I., Gruber M. (2008). Age and origin of major Smith-Lemli-Opitz syndrome (SLOS) mutations in European populations. *Journal of Medical Genetics*.

[17] Elbashir S. M., Harborth J., Lendeckel W., Yalcin A., Weber K., Tuschl T. (2001). Duplexes of 21-nucleotide RNAs mediate RNA interference in cultured mammalian cells. *Nature*.

[18] Harborth J., Elbashir S. M., Bechert K., Tuschl T., Weber K. (2001). Identification of essential genes in cultured mammalian cells using small interfering RNAs. *Journal of Cell Science*.

[19] Davies J. A., Ladomery M., Hohenstein P. (2004). Development of an siRNA-based method for repressing specific genes in renal organ culture and its use to show that the Wt1 tumour suppressor is required for nephron differentiation. *Human Molecular Genetics*.

[20] Sakai T., Larsen M., Yamada K. M. (2003). Fibronectin requirement in branching morphogenesis. *Nature*.

[21] Roux C., Wolf C., Mulliez N. (2000). Role of cholesterol in embryonic development. *The American Journal of Clinical Nutrition*.

[22] Li Y.-X., Yang H.-T., Zdanowicz M. (2007). Fetal alcohol exposure impairs hedgehog cholesterol modification and signaling. *Laboratory Investigation*.

[23] Battaile K. P., Battaile B. C., Merkens L. S., Maslen C. L., Steiner R. D. (2001). Carrier frequency of the common mutation IVS8-1G>C in DHCR7 and estimate of the expected incidence of Smith-Lemli-Opitz syndrome. *Molecular Genetics and Metabolism*.

[24] Setó-Salvia N., Stanier P. (2014). Genetics of cleft lip and/or cleft palate: association with other common anomalies. *European Journal of Medical Genetics*.

[25] Leslie E. J., Marazita M. L. (2013). Genetics of cleft lip and cleft palate. *American Journal of Medical Genetics Part C: Seminars in Medical Genetics*.

[26] Slavkin H. C. (1984). Morphogenesis of a complex organ: vertebrate palate development. *Current topics in Developmental Biology*.

[27] Thesleff I., Vaahtokari A., Partanen A.-M. (1995). Regulation of organogenesis: common molecular mechanisms regulating the development of teeth and other organs. *International Journal of Developmental Biology*.

[28] Francis-West P., Ladher R., Barlow A., Graveson A. (1998). Signalling interactions during facial development. *Mechanisms of Development*.

[29] Hu D., Helms J. A. (1999). The role of Sonic hedgehog in normal and abnormal craniofacial morphogenesis. *Development*.

[30] Barlow A. J., Francis-West P. H. (1997). Ectopic application of recombinant BMP-2 and BMP-4 can change patterning of developing chick facial primordia. *Development*.

[31] Wang Y.-H., Rutherford B., Upholt W. B., Mina M. (1999). Effects of BMP-7 on mouse tooth mesenchyme and chick mandibular mesenchyme. *Developmental Dynamics*.

[32] Koide T., Hayata T., Cho K. W. Y. (2006). Negative regulation of Hedgehog signaling by the cholesterogenic enzyme 7-dehydrocholesterol reductase. *Development*.

[33] Megha, Bakht O., London E. (2006). Cholesterol precursors stabilize ordinary and ceramide-rich ordered lipid domains (lipid rafts) to different degrees: implications for the bloch hypothesis and sterol biosynthesis disorders. *The Journal of Biological Chemistry*.

[34] Bitgood M. J., McMahon A. P. (1995). Hedgehog and Bmp genes are coexpressed at many diverse sites of cell-cell interaction in the mouse embryo. *Developmental Biology*.

[35] Chiang C., Litingtung Y., Lee E. (1996). Cyclopia and defective axial patterning in mice lacking Sonic hedgehog gene function. *Nature*.

[36] Lyons K. M., Pelton R. W., Hogan B. L. M. (1990). Organogenesis and pattern formation in the mouse: RNA distribution patterns suggest a role for Bone Morphogenetic Protein-2A (BMP-2A). *Development*.

[37] Zhang Z., Song Y., Zhao X., Zhang X., Fermin C., Chen Y. (2002). Rescue of cleft palate in Msx1-deficient mice by transgenic Bmp4 reveals a network of BMP and Shh signaling in the regulation of mammalian palatogenesis. *Development*.

